# Study on coupled mode flutter parameters of large wind turbine blades

**DOI:** 10.1038/s41598-024-62404-5

**Published:** 2024-06-04

**Authors:** Yong Zhuang, Guangming Yuan

**Affiliations:** School of Mechanical Engineering, Shandong University of Technology, Zibo, China

**Keywords:** Coupling mode, Flutter velocity, Flutter frequency, Parameter changes, Wind energy, Mechanical engineering

## Abstract

As the size of wind turbine blades increases, the flexibility of the blades increases. In actual operation, airflow flow can cause aerodynamic elastic instability of the blade structure. Long blades may experience coupled mode flutter due to the bending torsion coupling effect, leading to blade failure. Based on Euler Bernoulli beam theory combined with Theodorsen non directional aerodynamic loads, a blade flutter characteristic equation is established through finite element method. Taking NREL 5 MW wind turbine blades as an example, analyze the influence of parameter changes in different regions of the blades on flutter characteristics. Research has found that paramter changes in the tip region of blade have the greatest impact on flutter characteristics. The vibration frequency shows an overall upward trend with the increase of waving stiffness and torsional stiffness. The flutter velocity of the three regions tends to stabilize as the bending stiffness decreases. The blade flutter speed increases with the increase of torsional stiffness. The radius of gyration is inversely proportional to the flutter frequency and flutter velocity. The impact of centroid offset on blade structure flutter frequency is minimal, but the centroid offset in the tip region has a greater impact on flutter velocity. Increasing the torsional frequency can prevent coupled mode flutter and provide a theoretical basis for blade flutter prevention design.

## Introduction

Increasing the size of blades can improve the operational efficiency of wind turbines and also make them prone to aeroelastic instability problems^1–3^. Wind turbine blades are flexible blades with high aspect ratios, and blade flexibility can lead to bending torsion coupled flutter. Research on blade flutter characteristics^4–6^ has found that the aerodynamic elastic stability of blades is greatly affected by stiffness coupling. Pourazarm et al^7^ conducted a study: while keeping the torsional natural frequency constant, an increase in blade waving natural frequency leads to a decrease in flutter frequency; aeroelastic stability is greatly affected by the torsional natural frequency. Reduce the torsional natural frequency, and flutter will occur below the rated speed. Stablein et al^8^ analyzed the aerodynamic elastic modal characteristics and stability limits of a DTU 10MW wind turbine. Stablein et al^9^ conducted numerical simulations on the airfoil section at 75% of the blade span of a 10MW benchmark wind turbine, and pointed out that structural coupling mainly affects the frequency and damping of the coupled modes. Compared with uncoupled blades, blades with pendulum torsional coupling exhibit pendulum torsional flutter instability at lower inflow velocity. The problem of blade aeroelasticity is the study of the aerodynamic and structural models of blades. The beam element coupled aerodynamic model is the most widely used model for the aeroelastic stability of horizontal axis wind turbine blades, and the main process of aeroelastic analysis through the beam model^10^. Reference^11^ used a vortex model to simulate the wake of a dual blade wind turbine rotor. Due to the inherent singularity of the vortex plane in the wake, the vortex model is prone to divergence, which limits its application in wind turbine blades. The CFD method can iteratively solve the Navier Stokes equations for fluid flow control at thousands of positions around the blade, achieving visualization of fluid flow around the blade^12^. However, the process of establishing three-dimensional models in CFD methods is complex and not conducive to practical engineering applications. Theodorson unsteady aerodynamics is a method of calculating aerodynamic loads using two-dimensional oscillating airfoils. Claudia^13^ compared the unsteady vortex lattice, Euler method, and Reynolds averaged Naiver-Stokes method using Theodorsen theory to study the critical flutter velocity and limit cycle oscillation of two-dimensional airfoils under nonlinear conditions. It was pointed out that low fidelity methods can achieve the same phenomenon and reduce calculation time.

Reference^14^ points out that the flutter instability of wind turbine blades is mainly caused by the coupling of the waving bending mode and the twisting mode. For the airfoil sections of megawatt wind turbine blades in different regions, the stiffness in the swing direction is much greater than that in the waving direction^15^. Using NREL 5MW wind turbine blades as reference blades and based on one-dimensional beam model and Theodorsen aerodynamic model, this study investigates the influence of changes in blade waving stiffness, torsional stiffness, mass moment, center of gravity deviation, and other parameters on blade flutter. Using the Hamiltonian principle to derive the partial differential equation of a continuous beam coupled with bending and torsion, combined with Theodorson unsteady aerodynamic loads, solve the complex eigenvalue equation, and obtain the flutter frequency and velocity at the time of blade flutter occurrence. Using the Hamiltonian principle to derive the partial differential equation of a continuous beam coupled with bending and torsion, combined with Theodorson unsteady aerodynamic loads, solve the complex eigenvalue equation, and obtain the flutter frequency and velocity at the time of blade flutter occurrence. Divide the blade into three regions and study the influence of parameter variations in different regions on the coupled mode flutter characteristics. By analyzing the parameter changes in different regions, guide the aerodynamic elastic design of blades and provide theoretical basis for preventing blade flutter.

## Aeroelastic model of blades

The aeroelastic model consists of two parts: a structural model and an aerodynamic model. In structural models, Euler Bernoulli beams are often used for modeling, and the blade motion equations are established based on the Hamiltonian principle; and the unsteady aerodynamic loads are calculated using the Theodorson theory.

### Structural model

**Figure 1:**
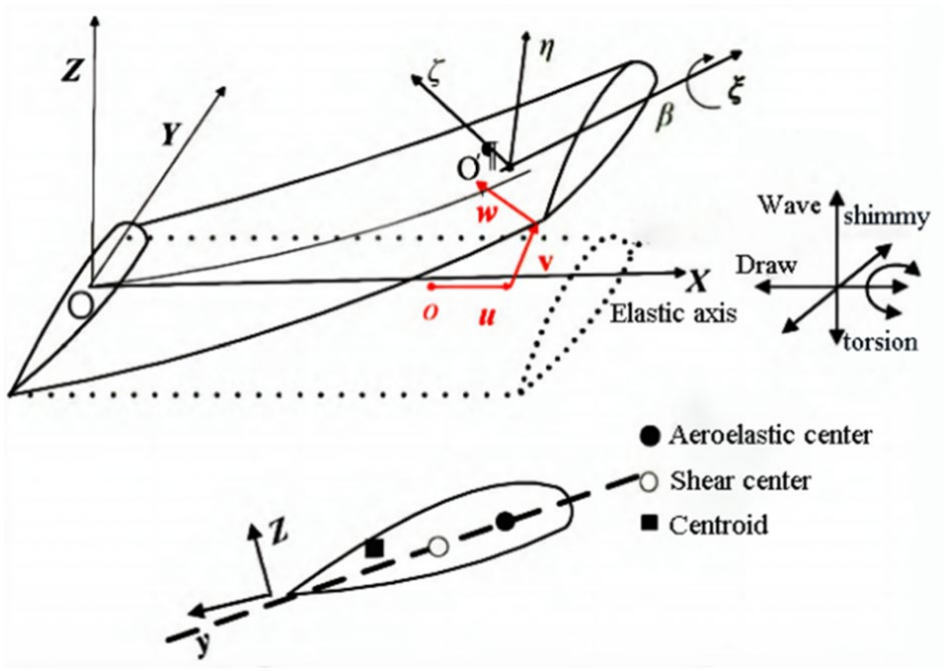
Blade Coordinate System.

Assuming that the blade main beam is an isotropic beam, based on the Euler beam motion differential equation derived by Hodges^16^, the structural model is shown in Fig. 1. The blade rotates at a constant angular velocity, and the blade root is fixed at the origin *O*. Establish two sets of coordinate systems: the overall blade rotation coordinate system *XYZ*, with the *x*-axis along the length direction and coincident with the elastic axis of the blade when it is not deformed, the *y*-axis in the rotation plane, and the *z*-axis perpendicular to the rotation plane. The local coordinate system $\xi \eta \zeta$ is fixed on the blade, $\xi$ is tangent to the elastic axis of deflection, and $\eta$ and $\zeta$ are the main coordinate axes after the deformation of the blade cross-section. *O* is a point on the elastic axis before deformation, $O^{\prime }$ is a point on the elastic axis after deformation, and *u*, *v*, and *w* are the displacements in the axial, waving, and oscillation directions of the blade. $\beta$ represents the geometric pitch angle of the blade.

The blade structure model includes two directions of motion: wave bending and twisting. The blade motion equation is established based on the Hamiltonian principle, as shown in the equation:

1 $$\mathop \smallint \limits_{{t_{1} }}^{{t_{2} }} \left( {\delta U - \delta T - \delta W} \right)dt = 0$$

$\delta U$ represents the variation of kinetic energy, $\delta T$ represent the variation of strain energy, and $\delta W$ represents virtual work. If only wave bend and torsion is considered, the differential equation of blade control can be expressed as:

2 $$L = \left[ {EIw^{`\text{'}} - e\phi \mathop \smallint \limits_{x}^{R} \Omega^{2} \rho Axdx} \right]^{\prime \prime } - \left( {w^{\prime } \left( {\mathop \smallint \limits_{x}^{R} \Omega^{2} \rho Axdx} \right)} \right)^{\prime } - \left( {\Omega^{2} mxe\phi } \right)^{\prime } + m\mathop w\limits + me\mathop \phi \limits$$

3 $$M = - \left[ {\left( {GJ + K_{m}^{2} \mathop \smallint \limits_{x}^{R} \Omega^{2} \rho Axdx} \right)\phi^{\prime } + } \right]^{\prime } + \Omega^{2} \rho A\left( {K_{{m_{2} }}^{2} - K_{{m_{1} }}^{2} } \right)\phi + \rho AK_{m}^{2} \mathop \phi \limits - e\left( {\mathop \smallint \limits_{x}^{R} \Omega^{2} \rho Axdx} \right)w^{\prime \prime } + \Omega^{2} mxew^{\prime } + me\mathop w\limits$$

Boundary condition settings:

4 $$x = 0,w = 0,w^{\prime } = 0,\phi = 0, x = L,w^{\prime \prime } = 0,w^{\prime \prime \prime } = 0,\phi^{\prime } = 0$$

where *L* and *M is* aerodynamic forces and variable pitch; *EI* represents the waving bending stiffness, *GJ* represents the torsional stiffness, and *w* and $\phi$ represent the displacement in the waving and twisting directions, respectively; *e* is the offset of the centroid; $\Omega$ is the speed of the wind turbine; $\rho$ is the leaf density; *A* is the cross-sectional area of the blade; *m* is the mass of the blade per unit length; ${K}_{m}$ is the polar radius of gyration around the elastic axis, and ${K}_{{m}_{1}}$ and ${K}_{{m}_{2}}$ are the axis mass radius of gyration around the main neutral axis and perpendicular to the elastic axis, respectively.

### Theodorsen aerodynamics

**Figure 2:**
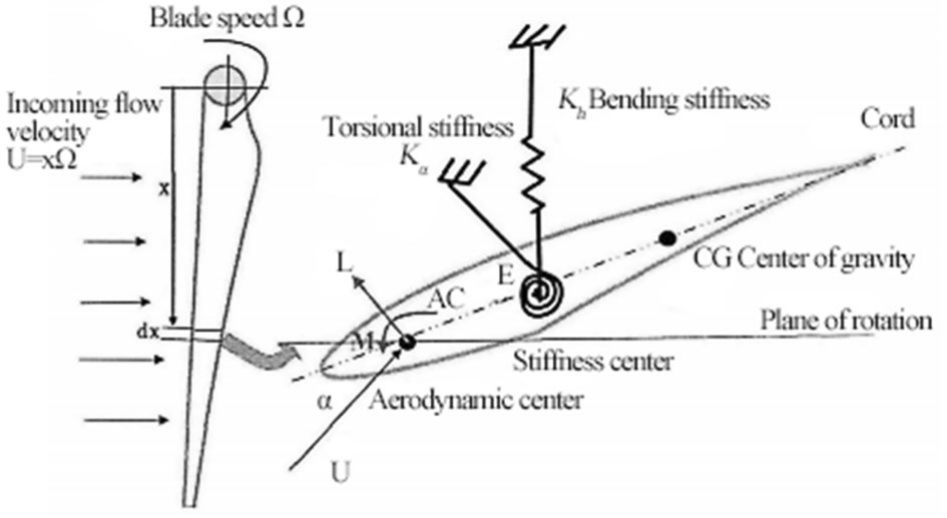
Aerodynamics on blade elements under bending torsion coupling mode.

As shown in Fig. 2, the three-dimensional blade rotates at an angular velocity $\Omega$, assuming that the blade rotates in stationary air, the incoming flow velocity is controlled by the blade rotation. When blade flutter occurs,the airflow effectively adheres to the surface of the blade. Assuming that the initial twist angle of the blade is zero, without considering the nonlinear term and the warping effect of the blade, it is assumed that the center of mass position of the rotating beam does not coincide with the elastic axis. AC is the aerodynamic center, E is the elastic axis, and CG is the center of gravity axis. The coupling form of blade bending and torsion degrees of freedom can be described by the two-dimensional airfoil's two degree of freedom coupled vibration model in the figure.

Theodorsen theory^17^ uses the potential flow assumption and Kutta condition to determine the aerodynamic forces of oscillating airfoils. The Theodorsen function $C(k)$ is a complex function based on the reduce frequency *k*, which can be transformed into a quasi-static aerodynamic model by transforming the function into an identity matrix. Lift *L* and variable pitch *M* can be expressed as:

5 $$L = - C_{L\alpha } \rho_{\infty } \left\{ {bC\left( k \right)U\dot{w} - \left[ {1 + C\left( k \right)\left( {1 - 2a} \right)} \right]\frac{{b^{2} }}{2}U\dot{\phi } - bC\left( k \right)U^{2} \phi + \frac{{b^{2} }}{2}\mathop w\limits + \frac{{ab^{3} }}{2}\mathop \phi \limits } \right\}$$

6 $$M = - C_{L\alpha } \rho_{\infty } \left\{ {b^{2} C\left( k \right)\left( {\frac{1}{2} + a} \right)U\dot{w} + \left[ {1 - C\left( k \right)\left( {1 + 2a} \right)} \right]\left( {\frac{1}{2} - a} \right)\frac{{b^{3} }}{2}U\dot{\phi } - b^{2} C\left( k \right)U^{2} \left( {\frac{1}{2} + a} \right)\phi + \frac{{ab^{3} }}{2}\mathop w\limits + \frac{{b^{4} }}{2}\left( {\frac{1}{8} + a^{2} } \right)\mathop \phi \limits } \right\}$$

where $C_{L\alpha }$ represents the lift coefficient; $\rho_{\infty }$ represents the air density; ${\text{reduce frequency }}k,$ where $k = \frac{{2\pi f_{{\text{f}}} b}}{u}$ and *f* is the flutter frequency; *b* is the half chord length; *a* is the distance from the elastic axis to the midpoint of the chord; *U* is the incoming flow velocity.

## Calculation and model validation of NREL 5MW blade flutter speed

Substitute Eqs. (5) and (6) into Eqs. (2) and (3), use finite element method and treat the blade as a variable cross-section linear Euler Bernoulli beam, with three degrees of freedom: $w,\delta \omega /\delta x,\phi$ at each node. The variational form of Eqs. (2) and (3) requires the interpolation function of the unit to be continuous, while the first derivative of $\phi$ and the second derivative of $w$ are non-zero. The structural region is discretized into a 1-dimensional 2-node element, assuming that the $\phi \left( x \right)$ and $w\left( x \right)$ of each node's degrees of freedom are linear functions and cubic polynomials, respectively. The displacement of unit nodes satisfies:

7 $$d_{{2\left( {i - 1} \right) + 1}}^{e} = w_{i}^{e} ,d_{2i}^{e} = \left( {\frac{\partial w}{{\partial x}}} \right)_{i}^{e} ,\phi_{i}^{e} \left( {i = 1,2} \right)$$

Assuming the displacement shape function is $w\left( x \right) = \mathop \sum \limits_{i = 1}^{4} N_{i}^{e} d_{i}^{e}$ and $\phi \left( x \right) = \mathop \sum \limits_{i = 1}^{2} P_{i}^{e} \phi_{i}^{e}$. Using the Galerkin method^18^, the second-order ordinary differential equation of the blade coupled beam element is obtained by weighted integration on the element:

8 $$\left[ {M_{s}^{e} + M_{a}^{e} } \right]\left\{ {\begin{array}{*{20}c} {\mathop w\limits } \\ {\mathop \phi \limits } \\ \end{array} } \right\}^{e} + \left[ {C_{a}^{e} } \right]\left[ {\begin{array}{*{20}c} {\dot{w}} \\ {\dot{\phi }} \\ \end{array} } \right]^{e} + \left[ {K_{s}^{e} + K_{a}^{e} } \right]\left\{ {\begin{array}{*{20}c} w \\ \phi \\ \end{array} } \right\}^{e} = \left\{ {\begin{array}{*{20}c} 0 \\ 0 \\ \end{array} } \right\}$$

Among them, ${M}_{s}^{e}$ and ${M}_{s}^{e}$ are the structural mass matrix and aerodynamic mass matrix; ${C}_{a}^{e}$ represents the aerodynamic damping matrix; ${K}_{s}^{e}$ and ${K}_{a}^{e}$ represents the structural stiffness matrix and aerodynamic stiffness matrix, respectively. Equation (8) can solve the complex eigenvalue problem through iterative methods to obtain the flutter frequency and damping of the blade. Calculate the damping ratio by dividing the negative real part of the complex eigenvalue by the modulus. When the damping ratio is zero, it indicates the occurrence of flutter. Define the frequency and velocity of flutter as flutter frequency and flutter velocity. The flutter frequency occurs at the moment of coupling between the first torsional and third bending modes, at which point the aerodynamic damping ratio corresponding to the flutter velocity is zero.

Using the airfoil parameters and section stiffness characteristics of NREL 5 MW blades in reference^19^, a MATLAB finite element flutter analysis program was developed to calculate blade flutter frequency and velocity. To verify the correctness of the program design, the natural frequency of the blade was calculated and compared with existing literature, as shown in Table 1. From the table, it can be seen that the program design is correct.

**Table 1:** Blade natural frequencies.

Modal ${ / }Hz$	Article	^14^	^7^	^19^	^20^
First	0.67	0.69	0.64	0.69	0.72
Second	1.96	1.8	1.86	2.02	2.05
Third	4.61	3.6	4.24	–	4.37
Fourth	5.71	8	5.39	–	5.62

To verify the correctness and reliability of the aeroelastic model of wind turbine blades, the blade flutter frequency and velocity were calculated and compared with existing literature models, as shown in Table 2. From the table, it can be seen that the calculated results are close to those in reference^7^.

**Table 2:** Comparison of flutter speed and frequency with existing research.

Types	Article	^14^	^7^	^21^	^16^
Speed ${ / }r \cdot m^{ - 1}$	21.36	24	20.7	26.4	19.1
Frequency ${ / }Hz$	4.7	4.1	3.6	5.43	3.4

## The influence of blade structural parameters on flutter

**Figure 3:**
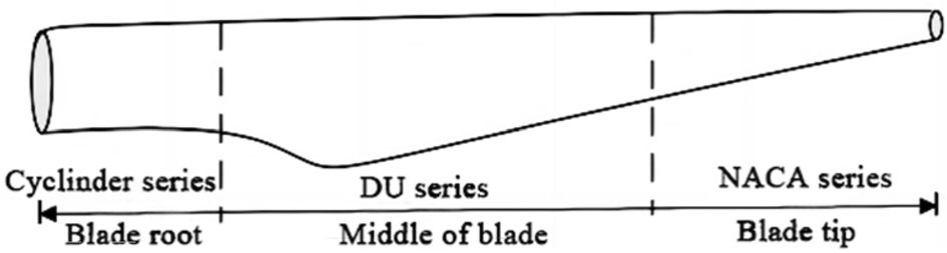
Three Areas of the Blade.

The aerodynamic elastic instability phenomenon of wind turbine blades belongs to modal coupling. When the first torsional mode of the blade is coupled with a certain waving mode, the blade occurs classical flutter. When the angle of attack and wind speed are the same, the flutter frequency of the blade will change with the variation of structural parameters. Large wind turbine blades have different airfoil cross-sections along the spanwise direction, and each airfoil has different composite material layers on the beam cap, web, leading edge, and trailing edge. Divide the wind turbine blades into three regions according to different airfoils, as shown in Fig. 3.

Assuming that the geometric shape of the blade and the aerodynamic forces acting on the blade remains unchanged, and considering the special characteristics of the airfoil section of the blade, a comparative study is conducted on the effects of structural parameter changes in different regions on the coupled modal flutter characteristics from the aspects of blade bending stiffness, torsional stiffness, center of mass displacement, and radius of gyration.

### The influence of waving stiffness

The classic manifestation of blade flutter is the coupling of bending mode and torsion mode, so the blade waving stiffness has a significant impact on the frequency of the bending dominated mode. Define the non dimensional waving stiffness as:

9 $$EI^{*} = \frac{{EI_{{\text{p}}} }}{{EI_{{\text{o}}} }}$$

Among them, $EI_{{\text{p}}}$ is the cross-sectional waving stiffness of the blade after changes, and $EI_{{\text{o}}}$ is the cross-sectional waving stiffness of the original blade. Simplify the blade as a cantilever structure, which can be seen as composed of upper and lower beam caps and front and rear web plates, with no bending torsion coupling effect on the front and rear web plates^22^. According to the theory of cantilever beams, Eq. (10) holds.

10 $$\omega_{F} \propto \sqrt {\frac{EI}{{\rho AL^{4} }}}$$

**Figure 4:**
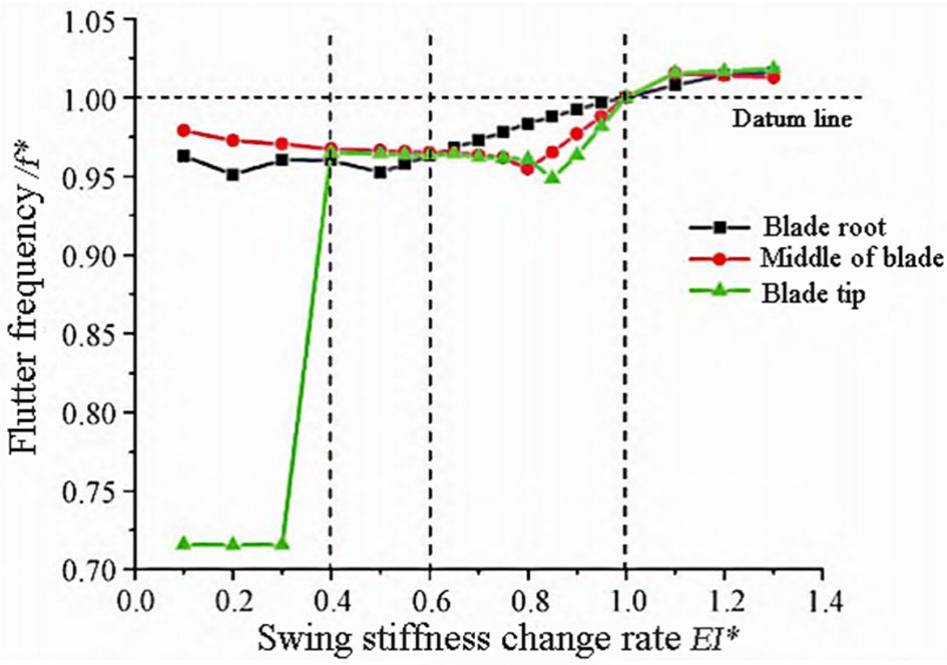
Flutter frequency variation with waving stiffness.

**Figure 5:**
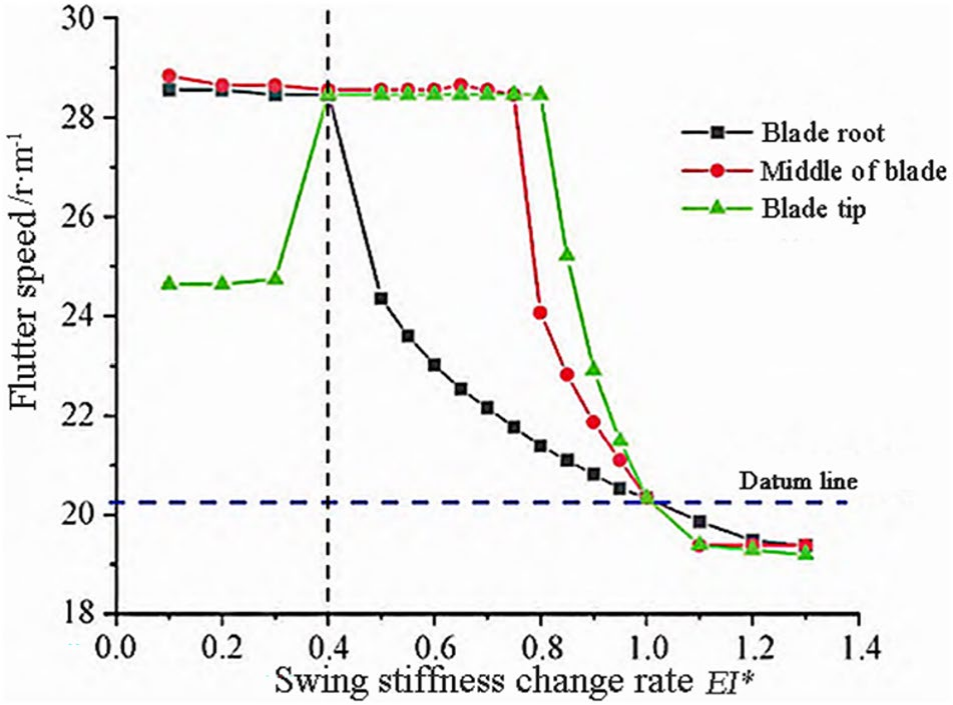
Flutter speed changes with waving stiffness.

According to Eq. (10), as the bending stiffness of the blade decreases and the mass and length of the blade increase, the flutter frequency of the blade will decrease. The variation trends of flutter frequency and flutter speed are shown in Figs. 4 and 5. From Fig. 4, it can be seen that when $E{I}^{*}<0.4$, the stiffness change in the blade tip region has a significant impact on the flutter frequency, reducing it by 30%. The blade tip flutter frequency is about 71% of the reference frequency. When $0.4<E{I}^{*}<0.6$, the stiffness change in the blade root area has a significant impact on the flutter frequency, reducing by about 5%. When $0.6<E{I}^{*}<1$, the change rule of the tip and middle regions of the blade is similar, with a maximum reduction rate of about 6% at the tip. When $E{I}^{*}>1$, the trend of changes in the three regions tends to be consistent, with the tip and middle regions of the blade having a significant impact on the flutter frequency.

As shown in Fig. 5, the frequency of the wave mode decreases with the decrease of bending stiffness, and the coupling time with the torsional mode is delayed. When $E{I}^{*}<1$, the flutter speed increases relative to the reference speed. When $E{I}^{*}<0.4$, the influence of the tip region is significant, and the trend of changes in the middle and root regions of the leaves is consistent. When $0.4<E{I}^{*}<0.8$, the changes in the tip and middle regions of the blade have a relatively small impact on the flutter speed, while the changes in the root region have a significant impact on the flutter speed. When $0.8<E{I}^{*}<1$, the blade flutter speed decreases to the reference speed as the stiffness increases, and the stiffness continues to increase. The flutter speed slowly decreases and tends to stabilize.

### The influence of torsional stiffness

The dimensionless torsional stiffness is defined as:

11 $${\text{G}}I^{*} = \frac{{GI_{{\text{p}}} }}{{GI_{{\text{o}}} }}$$

From equation (11), it can be concluded that:

12 $$\omega_{T} \propto \sqrt {\frac{GJ}{{\rho AL^{2} K_{m}^{2} }}}$$

**Figure 6:**
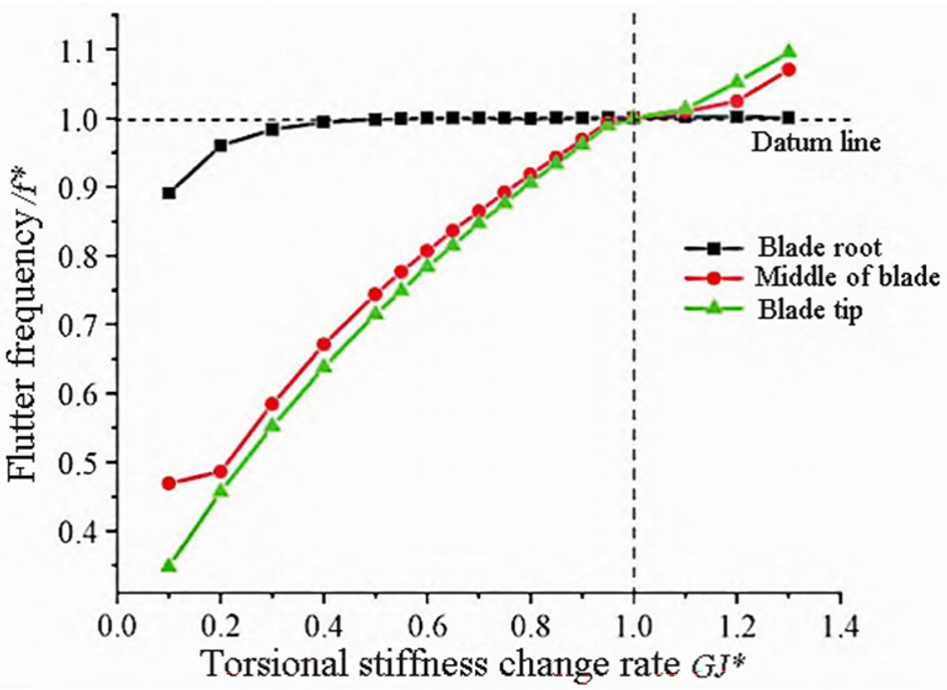
Flutter frequency variation with torsional stiffness.

**Figure 7:**
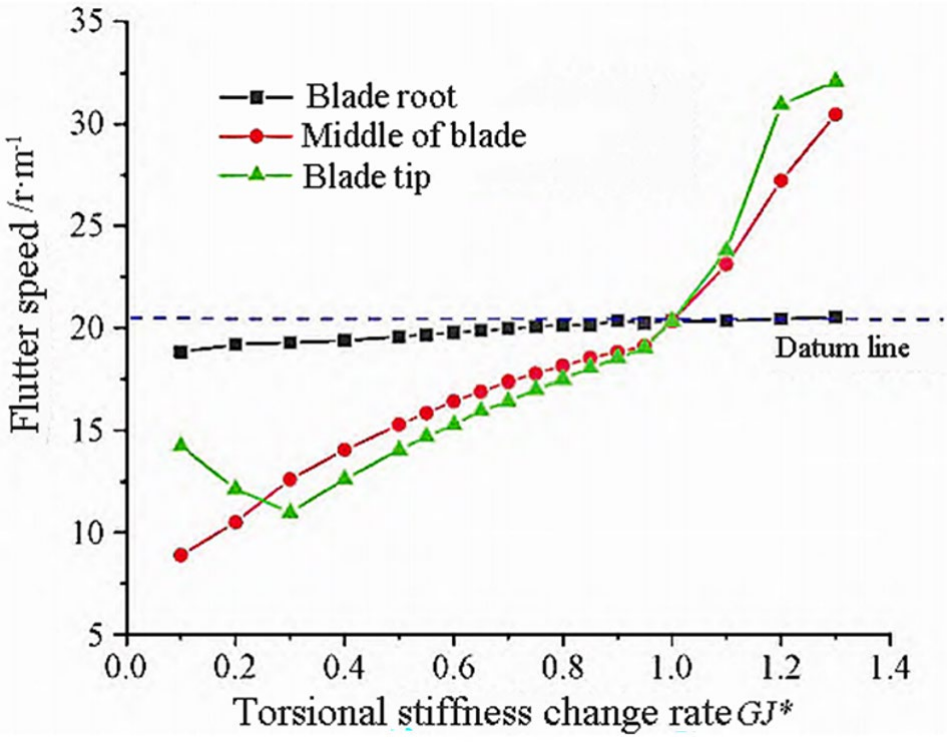
Flutter speed variation with torsional stiffness.

The torsional frequency of the blade decreases with the decrease of torsional stiffness, and is no longer coupled with the torsional and third-order wave modes, but rather coupled with the torsional and second-order or first-order wave modes to produce flutter. The variation trends of flutter frequency and flutter speed are shown in Figs. 6 and 7.

From Fig. 6, it can be seen that the blade flutter frequency decreases with the decrease of torsional stiffness, and the influence of the tip and mid blade regions on the flutter frequency is similar. From Fig. 7, it can be seen that the flutter speed in the tip and middle regions of the blade decreases rapidly with the decrease of torsional stiffness, while the decrease in the root region is slow. The torsional stiffness of the blade tip region has a significant impact on flutter velocity.

### The influence of centroid shift

The distance $e^{*}$ between the center of gravity and the elastic axis in the blade section is a dynamic instability parameter that affects the overall structure of the blade. The centroid position of the airfoil section of the blade is defined as the dimensionless centroid offset in Eq. (13):

13 $$e^{*} = \frac{{e_{{\text{p}}} }}{{e_{{\text{o}}} }}$$

**Figure 8:**
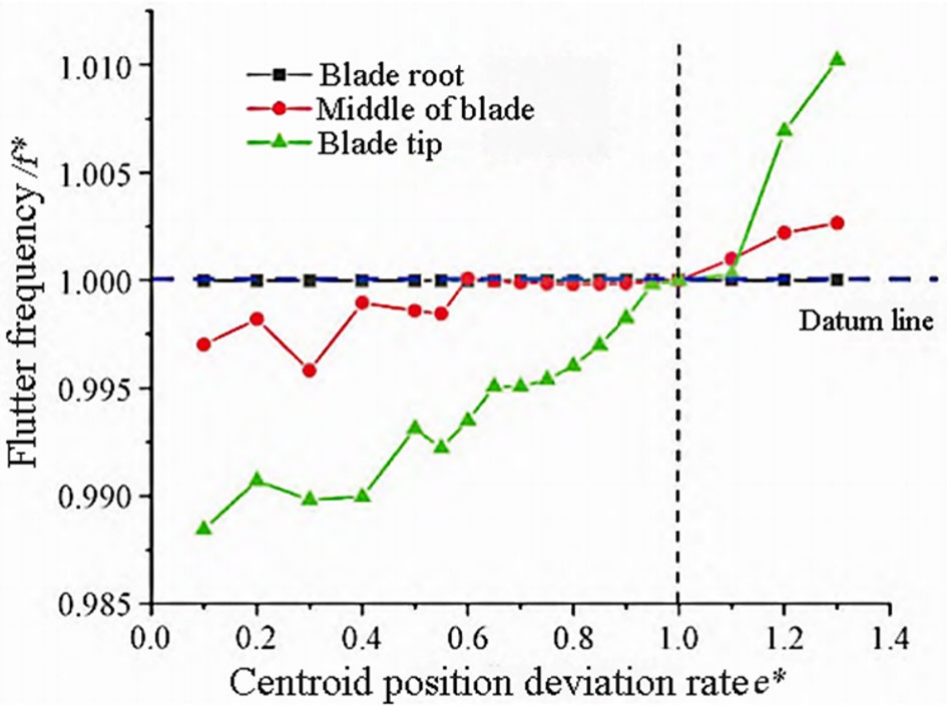
Flutter frequency variation with center of mass.

**Figure 9:**
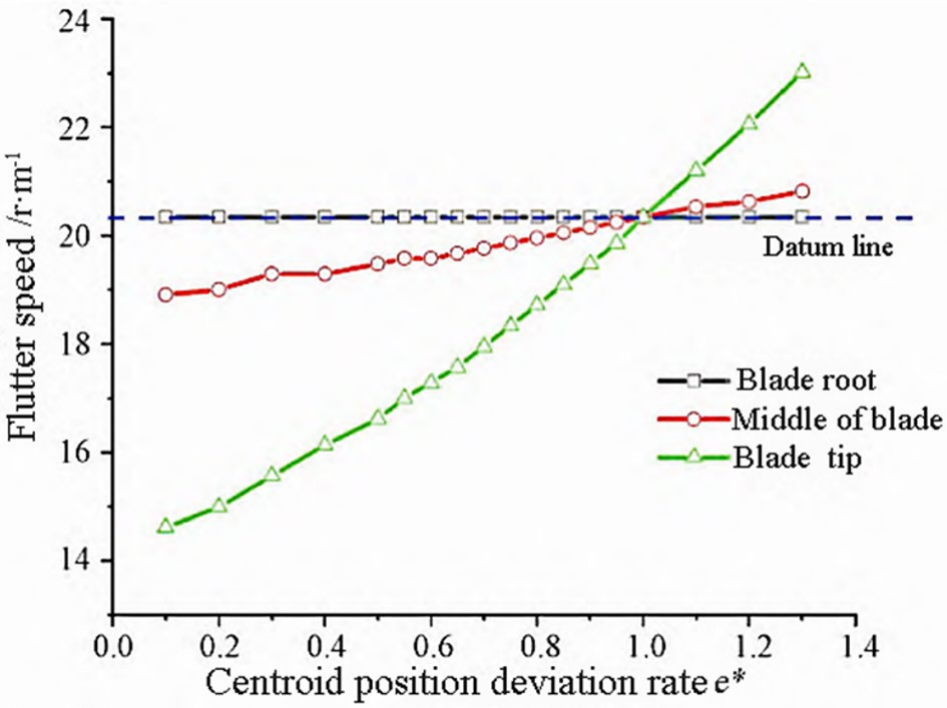
Flutter speed changes with center of mass.

Among them, $e_{{\text{p}}}$ and $e_{{\text{o}}}$ are the displacement of the center of mass of the changed blade and the reference blade, respectively. Flutter only occurs when the center of mass moves towards the trailing edge of the blade. The variation trends of flutter frequency and flutter speed are shown in Figs. 8 and 9.

From Fig. 8, it can be seen that the flutter frequency slightly increases with the increase of Centroid offset, and the influence of the blade tip egion is slightly greater. The maximum flutter frequency change rate is 2%. From the flutter speed curve in Fig. 9, it can be seen that the influence of the blade tip regin is bigger. As ${e}^{*}$ decreases, the center of mass moves backward towards the trailing edge of the blade, resulting in a decrease in flutter speed and a greater likelihood of flutter occurring.

### The influence of radius of gyration

The mass moment of the blade is inversely proportional to the square of the radius of gyration. Define the dimensionless radius of gyration of the blade with respect to the main neutral axis and the coordinate axis perpendicular to the chord that passes through the elastic axis:

14 $$Km_{1}^{*} = \frac{{Km_{1p} }}{{Km_{1o} }},Km_{2}^{*} = \frac{{Km_{2p} }}{{Km_{2o} }}$$

$K_{m}$ is the polar moment of inertia, as can be obtained from Eq. (12), the $K_{m}$ is inversely proportional to the torsional mode frequency. $K_{m1}$ and $K_{m2}$ are the radius of gyration, and their numerical values can reflect the mass distribution characteristics of the elastic axis of the blade cross-section, and the relationships between $K_{m}$, $K_{m1}$, and $K_{m2}$ meet the following requirements:

15 $$K_{m}^{2} = K_{m1}^{2} K_{m2}^{2} = \frac{1}{m}{\iint }\rho \left( {x^{2} + y^{2} } \right)dxdy$$

**Figure 10:**
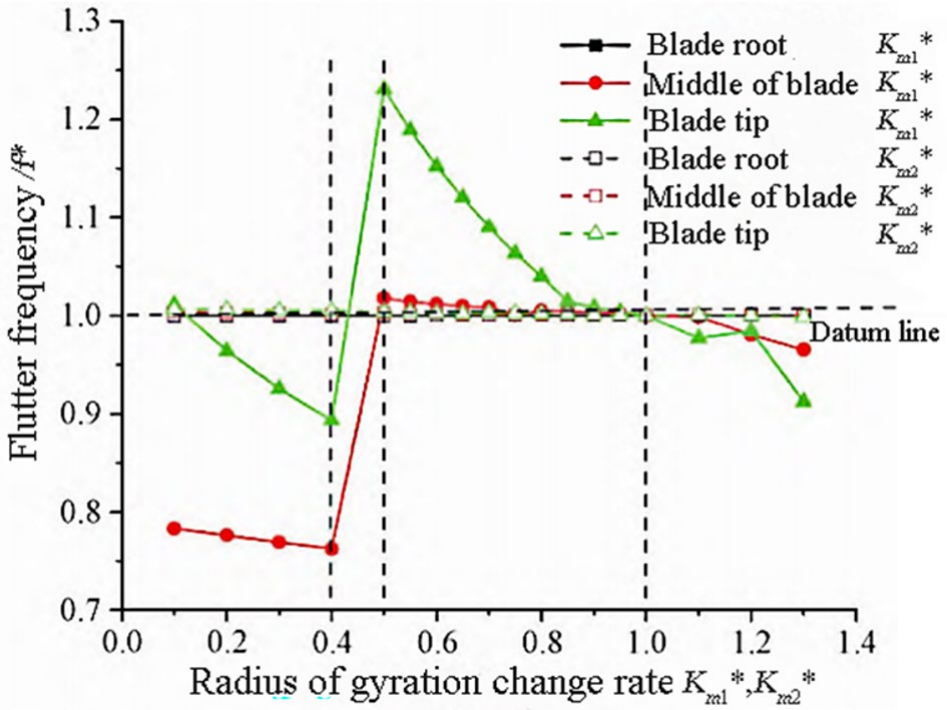
Flutter frequency variation with radius of gyration.

**Figure 11:**
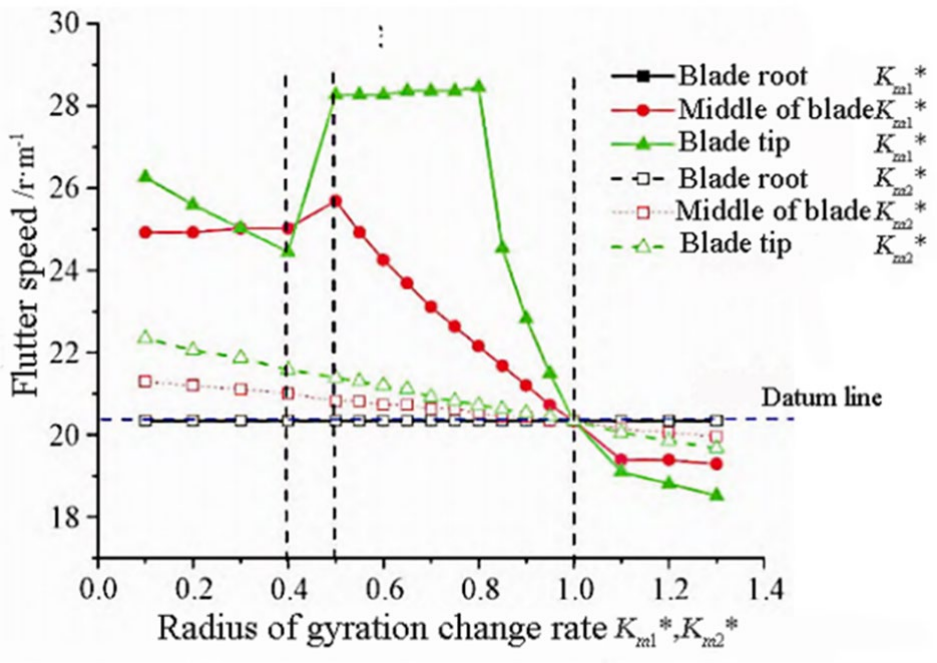
Flutter speed variation with turning radius.

The variation trends of flutter frequency and flutter speed are shown in Figs. 10 and 11. From Fig. 10, it can be seen that the radius of gyration $K{{m}_{1}}^{*}$ of the blade section on the *y*-axis has a significant impact on the flutter frequency. When $K{{m}_{1}}^{*}>0.5$, the blade flutter frequency decreases with the increase of the radius of gyration, indicating that the farther the mass distribution of the blade section is from the elastic axis, the more prone it is to flutter. When $K{{m}_{1}}^{*}<0.4$, the flutter frequencies in the tip and center regions of the blade decrease with the increase of the radius of gyration, and the overall flutter frequency is smaller than the reference frequency. When $K{{m}_{1}}^{*}=0.4$, the flutter frequency in the mid blade region reaches the minimum value, which is 0.76 times the reference frequency. When $K{{m}_{1}}^{*}=0.5$, the flutter frequency reaches its maximum value, which is 1.23 times the reference frequency.

As shown in Fig. 11, due to the influence of the radius of gyration, the trend of flutter speed change is similar to that of flutter frequency, and decreases with the increase of the radius of gyration $K{{m}_{2}}^{*}$. The influence of the cross-sectional turning radius $K{{m}_{1}}^{*}$ in the root region on the flutter speed is almost zero, while the influence of the cross-sectional turning radius in the tip and center regions on the flutter speed is complex. When $0.5<K{{m}_{1}}^{*}<0.8$, the flutter speed reaches its maximum value. When $K{{m}_{1}}^{*}>0.8$, the increase in the turning radius of the blade tip region causes a decreasing trend in flutter velocity. The radius of gyration is within the interval $\left[{0.4,0.5}\right]$, and there is a jumping instability phenomenon at the critical flutter speed.

## Conclusion

The blade is simplified as an Euler Bernoulli beam, and combined with the unsteady aerodynamic theory of Theodorson, the coupled flutter dynamic equation of the blade considering the Characteristics of coupled motion between waving and twisting. The blade flutter characteristic equation is solved using the complex eigenvalue method. The frequency and velocity of NREL 5 MW blade flutter was solved, and the correctness and reliability of the wind turbine blade aeroelastic model were verified by comparing it with existing literature models.

A study was conducted on the coupled modal flutter parameters of NREL 5 MW blades, and it was verified that the coupling of the first torsional mode and a certain wave bending mode of the blades can cause classical flutter phenomena. The research results indicate that:

- By analyzing the structural parameter changes in the blade tip, middle, and root regions, it was revealed that the parameter changes in the blade tip region have the greatest impact on flutter frequency and velocity.
- The flutter frequency shows an overall upward trend with the increase of waving stiffness and torsional stiffness. The flutter velocity of the three regions tends to stabilize as the bending stiffness decreases. The blade flutter speed increases with the increase of torsional stiffness. The radius of gyration is inversely proportional to the flutter frequency and flutter velocity. The impact of centroid offset on blade structure flutter frequency is minimal, but the centroid offset in the tip region has a greater impact on flutter velocity.
- By analyzing the parameter changes in different regions, guide the aerodynamic elastic design of blades and provide theoretical basis for preventing blade flutter. In the problem of blade flutter in megawatt wind turbines, wave frequency frequency and torsional frequency has become the main considerations for future design. In the design of blade anti flutter, the influence of torsional stiffness should be emphasized. How to maintain a high torsional frequency and avoid coupling with a certain bending mode has become a key factor in improving the flutter limit.

### Supplementary Information

Supplementary Information.

## Data Availability

All data generated or analysed during this study are included in this published article and its supplementary information files.
